# Diffusion tensor magnetic resonance imaging (DTI) tractography-guided deep brain stimulation in neuropathic pain

**DOI:** 10.1007/s00701-015-2356-1

**Published:** 2015-02-05

**Authors:** Volker A. Coenen, Kristin Kieselbach, Irina Mader, Peter C. Reinacher

**Affiliations:** 1 Department of Stereotactic and Functional Neurosurgery, Freiburg University Medical Centre, Breisacher Strasse 64, 79106 Freiburg i.Br., Germany; 2 Department of Neuroradiology, Freiburg University Medical Centre, Freiburg i.Br., Germany; 3 Interdisciplinary Pain Centre, Freiburg University Medical Centre, Freiburg i.Br., Germany

Dear Editor,

Chronic pain syndromes pose a challenge for interdisciplinary teams of pain specialists. We report a patient who presented with a neuropathic trigeminal pain syndrome after repeated resection of an epidermoid tumour involving the trigeminal ganglion. Multiple therapeutic approaches—including chronic motor cortex stimulation, intrathecal drug application and deep brain stimulation (DBS) to the periventricular/periaqueductal grey and sensory thalamus—did not lead to a sustained relief of pain with a persistent rating of 7-9 on the visual analogue scale (VAS). A magnetic resonance imaging (MRI) scan was suspicious for a malposition of the previously implanted clinically non-functional DBS electrodes. The DBS system was completely removed surgically. The patient underwent diffusion tensor magnetic resonance imaging (DTI). Imaging was performed on a clinical 3-T MRI system (Magnetom Trio Tim System 3 T; Siemens, Erlangen, Germany). DTI: single-shot, two-dimensional, SE EPI; TR, 10,000 ms; TE, 94 ms; diffusion values, *b* = 0 s/mm^2^ and *b* = 1,000 s/mm^2^; diffusion directions, 61; slice count, 69; voxel size, 2.0 × 2.0 × 2.0 mm^3^; acquisition time, 11:40 min. Deformation correction of the EPI sequence according to Zaitsev et al. [1]. DTI tractography: StealthViz-DTI system (Medtronic Navigation, Louisville, USA); FA level, 0.2; minimal fibre length, 10 mm; seed density, 5.0; maximal fibre cut-off angle, 50°. Tractography as shown here used the MCP coordinates of the previous (removed) and newly implanted electrodes. Three-dimensional visualisation and rendering of tracked fibres were performed with Amira (Konrad Zuse Zemtrum, Berlin, Germany and Visualization Sciences Group, SAS Bordeaux, France); electric stimulation as previously described [2].

At the day after imaging, two DBS electrodes were implanted stereotactically, assisted with the DTI technology (MCP coordinates cross-checked with DTI fibre-tracking results: VCP: laterality 11 mm, 2-mm anterior to PC, verticality at level of ACPC plane; PVG/PAG: 5-mm laterality, 2-mm anterior PC, verticality at level of ACPC plane).

**Fig. 1:**
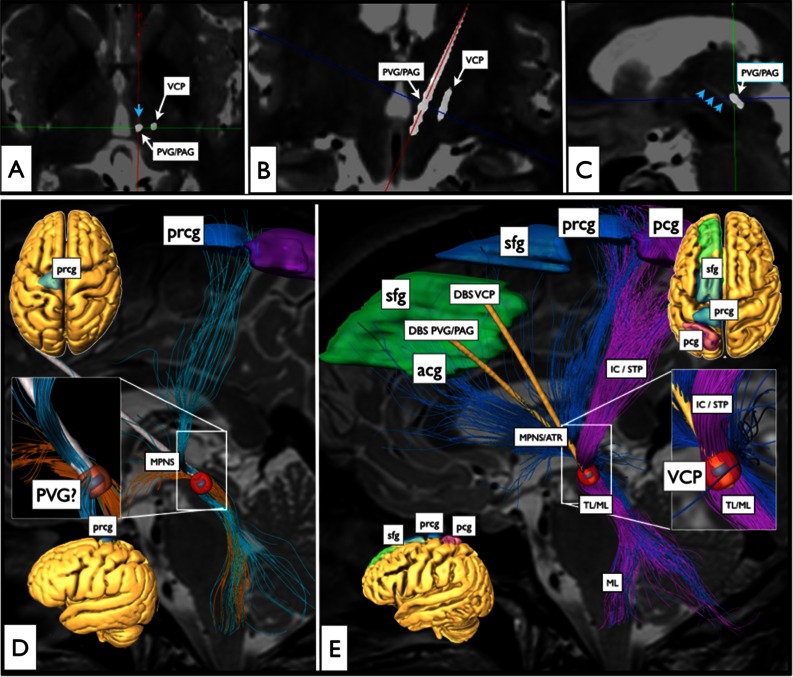
**a**–**c** Deep brain stimulation (DBS) electrode positions (postoperative computed tomography superimposed onto preoperative T2-weighted MRI scans): **a** axial view at the level of the inter-commissural plane; **b** coronal view; **c** parasagittal view. *Blue arrowheads* in **a** and **c** indicate previous implantation site of a DBS electrode (removed). A second electrode (from a previous operation, removed) was located intraventricularly, its tract site is not discernible. **d**, **e** Results from diffusion tensor imaging (DTI) fibre-tracking analysis combined with individual electric field simulation studies: **d** unsuccessful previous treatment with two DBS electrodes; **e** successful stimulation attempt over VCP and PAG DBS electrodes now reaching the medial (*blue* MPNS) and lateral (*pink* ML) systems (*acg* anterior cingulate gyrus [BA24], *ATR* anterior thalamic radiation, *IC/STP* internal capsule/superior thalamic peduncle, *MPNS* midline polysynaptic nociceptive system, *VCP* nucleus ventralis caudalis posterior, *PVG/PAG* periventricular grey/periaqueductal grey, *prcg* precentral gyrus, *pcg* postcentral gyrus, *sfg* superior frontal gyrus, *TL/ML* trigeminal lemniscus/medial lemniscus)

DTI tractography analysis revealed that the previously misplaced DBS electrodes were touching the median polysynaptic pain system (MPNS) (Fig. 1a-c, *blue arrows*). The newly placed electrodes (as displayed with helical computed tomography) now in addition reached the medial and trigeminal lemniscal systems (Fig. 1d, e). The patient’s VAS dropped almost instantaneously and remained stable with fluctuating levels between 2 and 5 over a period of 15 months.

Recently, DBS of the medial lemniscus in neuropathic pain after planning with tractography has been described [3]. This line of investigation is likely to be fruitful in the light of successful stimulation of fibre tracts in other indications [2, 4–6]. The PAG/MPNS system is concerned with autonomic, emotional and pain function. In the context of DBS, the connectivity of the PAG has been previously addressed utilising the DTI technology [7, 8]. In accordance with our own tractography studies [9], we are convinced that one previously described upstream projection [8] is confluent with the ATR system that at different levels (midbrain, PAG, prefrontal cortex, bed-nucleus of the stria terminalis) connects with the MFB system. Recently we have shown in a pilot study that direct stimulation of the MFB has strong anti-depressive effects [6]. Since PAG/PVG stimulation is located in a supposed connection hub of MFB/ATR, it is possible that PAG stimulation alters the network balance of the ATR system in favour of the MFB system and diminishes emotional and somatic pain. We have elaborated on the limitations of the DTI technology in our previous publications [2, 4, 5].

This report strengthens the idea of the application of the DTI tractography technology for DBS surgery in neuropathic and nociceptive pain and, moreover, for the thorough analysis of the electrode positions obtained with respect to the fibre systems, which in addition to computation of somatic pain are concerned with emotion processing.
